# Supplementary material for: The zoo of the gene networks capable of pattern formation by extracellular signaling
Source: eLife. 2026 Nov 13;14:RP107563. doi: 10.7554/eLife.107563 (PMC13766667; doi:10.7554/eLife.107563)
Supplement: Peer review history

# Editor report: eLife Assessment

**DOI:** 10.7554/eLife.107563.4.sa0

**Authors:** Ariel Amir

**Affiliations:** https://ror.org/0316ej306 Weizmann Institute of Science Israel

**Keywords:** Incomplete, Valuable

The study presents a **valuable** conceptual framework by classifying pattern-forming gene subnetworks into three established categories. By meticulously enumerating and simulating various network topologies, the authors provide a systematic catalog of these patterning motifs. However, the supporting evidence remains **incomplete**, as the mathematical generalizations rely on simplified assumptions that may not hold in more complex or realistic scenarios.

# Referee report: Reviewer #1 (Public review)

**DOI:** 10.7554/eLife.107563.4.sa1

Summary:

The authors tackle a long-standing question in developmental theory: given a gene-regulatory network that includes extracellular signaling, which topologies are even capable of transforming an initial spatial profile into a genuinely new pattern? Building on the classical reaction-diffusion framework in one dimension, but imposing biologically motivated constraints, they prove that every one-signal sub-network must be either Hierarchical (H), self-activating (L+), or self-inhibiting (L-). They further demonstrate that only three composite classes of full networks - pure H, a coupled L+ L- "Turing" pair, and an L- module fed by an intracellular positive loop ("noise-amplifying")-can create non-trivial spatial transformations. Analytical criteria and illustrative simulations are provided, together providing a closed taxonomy, which is supposed to be relevant for real systems.

Strengths:

- Useful classification framework. Reducing a vast number of possible gene circuits to three canonical pattern-forming motifs is a valuable organizing insight for both theorists and experimentalists.

- Practical interpretability. Given a reaction network diagram, one can now decide (assuming the model applies to real systems) whether spatial patterning is even possible, saving experimental effort on in silico screens that could never succeed.

Weaknesses:

- Theoretical limitations in the application of Linear Stability Analysis (LSA): I remain uncertain about the framework's reliance on LSA as a necessary condition for non-trivial pattern transformation, especially for large initial perturbations ("spikes"). The revised manuscript itself states that spike amplitudes must be sufficiently small for the linearization to hold. In the rebuttal, the authors argue that large spikes can nevertheless be treated because their influence is initially small outside the spike. However, linear stability of a homogeneous steady state only describes the response to infinitesimal perturbations around that state; it does not generally exclude finite-amplitude perturbations from entering a different nonlinear basin of attraction and producing a heterogeneous stationary state, e.g., as in subcritical Turing patterns. Thus, I do not think the rebuttal establishes the stronger claim that a linearly stable network cannot produce a non-trivial pattern regardless of nonlinear terms.

- Presentation: The manuscript remains difficult to follow. The argument is distributed across many named requirements and topology classes, long prose descriptions of network structures, and repeated cross-references to the Supplementary Information. Given that the main contribution is a conceptual classification, I think the logical hierarchy should be considerably easier to reconstruct.

Discussion:

The study offers a solid conceptual organization of pattern-forming networks. However, the theoretical bridge between infinitesimal linear stability and macroscopic, non-linear pattern emergence still presents some uncertainties. The way the current framework formally treats large initial perturbations leaves some questions open regarding its broad analytical applicability to real biological tissues.

# Author comment: Author response

**DOI:** 10.7554/eLife.107563.4.sa2

**Authors:** Kevin Martinez Anhom, Isaac Salazar-Ciudad

**Affiliations:** Institut Pasteur Paris France; Universitat Autònoma de Barcelona Cerdanyola del Valles Spain

The following is the authors’ response to the previous reviews.

**Public Reviews:**

**Reviewer #1 (Public review):**

Summary:

The authors tackle a long-standing question in developmental theory: given a gene-regulatory network that includes extracellular signaling, which topologies are even capable of transforming an initial spatial profile into a genuinely new pattern? Building on the classical reaction-diffusion framework in one dimension, but imposing biologically motivated constraints, they prove that every one-signal sub-network must be either Hierarchical (H), self-activating (L+), or selfinhibiting (L-). They further demonstrate that only three composite classes of full networks - pure H, a coupled L+ L- "Turing" pair, and an L- module fed by an intracellular positive loop ("noise-amplifying")-can create non-trivial spatial transformations. Analytical criteria and illustrative simulations are provided, together providing a closed taxonomy, which is supposed to be relevant for real systems.

Strengths:

(1) Useful classification framework. Reducing a vast number of possible gene circuits to three canonical patternforming motifs is a valuable organizing insight for both theorists and ---experimentalists.

(2) Practical interpretability. Given a reaction network diagram, one can now decide (assuming the model applies to real systems) whether spatial patterning is even possible, saving experimental effort on in silico screens that could never succeed.

Weaknesses:

(1) After the resubmission, I still have concerns regarding the formal definition of "non-trivial transformations" (P1/P2) and its application to noisy or multi-dimensional systems. The criteria rely on counting "new" critical points (maxima/minima). In their response, the authors argue that the diffusion operator instantly smooths discontinuous white noise, allowing critical points to be properly defined. However, this very smoothing process passively generates a landscape of new, smooth local extrema from the initial noise. Consequently, trivial diffusive regularization could inadvertently fulfil the criteria for a "non-trivial" transformation, leaving the definition conceptually problematic.

That is indeed the case: diffusion alone can generate new concentration maxima and minima; but these would be transient unless there are some self-activatory loops (as we detail over the article). If these concentration maxima and minima are transient in time, they do not count for pattern transformation. In the two version of the article we explicitly stated (in P1 in the introduction when defining pattern transformation) that we only consider resulting patterns that are stable in time. This implies that the concentration maxima and minima that may transiently arise from diffusion alone do not count for the definition of pattern transformation. In the current new version (the third) and after the reviewer’s suggestion, we insist (in red text) that the new concentration maxima and minima need to be stable in time (i.e. non-transient).

Furthermore, when extending the framework to 2D/3D, the manuscript assumes that starting from a central "spike" will robustly preserve radial symmetry, yielding concentric rings or shells. This overlooks the fundamental nature of macroscopic mean-field models like reaction-diffusion equations. The realization of the final multidimensional pattern depends strictly on the stability of the solution against ubiquitous perturbations (including angular modes) rather than solely on the deterministic symmetry of the initial condition. It remains unclear how the current framework accounts for spontaneous symmetry breaking in cases where these angular modes become unstable, challenging the assumption that radial symmetry will strictly dictate the outcome. We note that the authors' use of noise as an initial condition does not resolve this fundamental issue. Reaction-diffusion equations inherently describe mean-field dynamics, meaning that microscopic fluctuations are continuously present in any real system, regardless of whether explicit stochastic terms are written into the equations. Ultimately, if a symmetric mean-field solution is structurally unstable to these inherent fluctuations, it simply cannot be realized in nature.

If we understood right the reviewer is saying that there are always fluctuations everywhere and that, thus, the spike initial pattern should also have noise everywhere. In the discussion (and partially in the introduction) we have now added a discussion (in red) on how the resulting patterns possible from such spike-with-noise initial pattern can actually be understood, to a large extent, from those of the homogeneous-with-noise and spike-without noise. In that case, as the reviewer suggests, the resulting patterns do not necessarily have radial symmetry. We have kept the results on spike initial patterns without noise because they are very helpful to understand the pattern transformations possible from spike-with-noise initial patterns. Moreover, as we discuss now, the spatial fluctuations that occur everywhere can be really small compared to the concentration in the spike and, thus, the spike initial pattern without noise can be a good approximation in some cases, at least worth considering.

(2) Theoretical limitations in the application of Linear Stability Analysis (LSA): I remain uncertain about the framework's reliance on LSA to categorize macroscopic transformations, especially those arising from large initial perturbations (spikes). In their rebuttal letter, the authors justify this by assuming the perturbation remains small over a short time interval. However, because the study aims to describe stationary, asymptotic states, applying a linear approximation that relies on transient t->0 conditions to predict long-term global stability is not fully resolved.

We are not trying to predict long-term stability and we never intended to. We never claimed that to be the case. We are interested in stable in time spatial patterns but the long-term stability of resulting patterns is not something we intend to see from the LSA. The LSA just provides a necessary (but not sufficient) condition for non-trivial pattern transformations: any network unable to sustain the growth of small perturbations (i.e., any linearly stable network topology) cannot lead to a non-trivial pattern transformation, regardless of the nonlinear terms in the reaction term f(g). From the previous suggestions by the reviewer it could be the case that he/she thinks that since there is always noise everywhere and all the time we can never apply the LSA or that it cannot be applied along time. In fact, we do not try to applied over time (that would make no sense for our purposes). The LSA is only applicable and only informative when applied to the initial pattern (that is at time 0) to see gene network that cannot transform initial patterns into other patterns. As we discuss in the previous version and we further stress in the current one (in red in the LSA), large spikes do not invalidate this approach. Even if the spike would be large, it would only affect cells outside the spike through the diffusion of gene products from the spike. Thus, if a small enough time is considered, large spikes can be considered as small concentration perturbations outside the spike and, thus, in the the worse case scenario our whole approach may not be applicable inside the spike but outside of it (that is reasonable since spikes are by definition narrow).

Here it is important to stress that many things that used the LSA in the original version of the article do not used it in the current version of the article. In fact, in this article we address two main questions: (i) which gene network topologies can produce non-trivial pattern transformations; and (ii) what can we say about the stationary patterns they produce. We acknowledge that a linear stability analysis alone is not enough to fully characterize the long-term behavior of the system, and this is why we only use LSA as an aid to answer question (i).

This was not clear enough in the first version of the article but it was explicitly stated in the last version (in the Gene network classification and Linear stability analysis section). This allows us to discard many topologies, but further analysis is still required to assess whether linearly unstable network topologies can actually produce non-trivial pattern transformations.

It is in this further study that question (ii) comes to play. Here we do not rely on LSA, but instead impose a series of requirements (R1-R5) on the reaction term f(g) that constrain the nonlinear dynamics in a biologically motivated way that prevents pathological behaviors (particularly, boundedness of solutions (R4) and monotonicity of the reaction (R5)). These requirements enable the qualitative analysis of the different unstable network topologies in order to say some things about the possible stationary patterns. This is complemented with numerical simulations and quantitative analysis of some prototypical examples in the supplementary information.

(3) In the previous round of the review, I suggested that a biomolecular sink, such as A+B -> AB reaction, could break the approach. In their response letter, the authors defend their approach by arguing that such reactions can be accommodated by their abstract constraints (R1-R5) as long as the signs of the Jacobian elements remain invariant. However, the problem I see here is not the sign of the interactions, but the severe loss of spatial homogeneity.

When a macroscopic initial perturbation (a "spike" of morphogen) is introduced into a domain with a strong bimolecular sink, it will inevitably cause massive local depletion of the consumed substrate near the source. Consequently, the background state of the system will rapidly evolve into a profile with macroscopic spatial gradients long before any spontaneous pattern-forming instability takes over. Mathematically, this dictates that the system no longer possesses a homogeneous steady state, and the Jacobian matrix becomes explicitly space-dependent, which should break the classical LSA approach.

This criticism seems to be intimately related to the previous one. If we understand correctly, the argument is that in a very non-linear system, such as in the sink described, the spike will rapidly lead to a local change in the concentration of other gene products and that then the LSA is not applicable. This is true but what we care about is whether the initial pattern (that is the system at time zero) is actually stable or not. We care about it because as we explain, pattern transformation is only possible if the perturbation in the initial pattern (spike or noise) is unstable. This is simply a necessary condition (an initial pattern may be unstable to perturbation and still not produce nontrivially transformations). So whether the system will be suitable for a LSA some time after the initial pattern, as the reviewer suggests, is not something we need to know for our classification. Related to the other comments the reviewer may be concerned with whether other perturbations occurring everywhere (and all the time), that is noise, may actually affect the possible resulting patterns (that we described in the new section of the discussion).

We want to thank the reviewer for this clarification, as we believe we had not fully understood their concern in the previous round of review. In our framework, the bimolecular sink the reviewer suggests corresponds to a three-gene-product network where A and B mutually inhibit each other and both activate AB.

First it is important to consider that unless something else is specified an A+B→AB system with homogeneous initial pattern (with or without noise) is just not stable: the A and B gene products will decay to zero concentration and AB to a maximal concentration (that would be homogeneous over space if there is no noise). Besides the final stable state is totally stable since with no A or B left the concentration of AB cannot change. We explicitly state in the article (in the LSA section) that we apply the LSA to systems that, when unperturbed, are stable. For other systems we just wait for the system to stabilize and then ask whether pattern transformation is possible from that state if there is some perturbation, where we can now apply a LSA. So strictly speaking the system the reviewer is suggesting is outside the scope of the article and indeed unsuitable for LSA. Nevertheless, the system the reviewer proposes cannot lead to non-trivial pattern formation, as we detail below.

The reviewer does not specify whether A, B or AB diffuse, we then consider all possibilities. We also assume that A is the gene product in the spike.

Case in which no molecule diffuses. In this case a spike of A will simply lead to a valley of B (since A reacts with B to deplete B), and a spike of AB in the exact same location of the spike (i.e., no non-trivial pattern transformation occurs). If there is noise, each small fluctuation in the concentration of A or B would lead to a similar fluctuation in AB. Notice this case does not lead to non-trivial pattern transformations (the peaks in the initial pattern and resulting pattern are in the same places). This latter situation in fact we explain in the “Pattern formations from homogeneouswith-noise initial patterns in H networks section.”

Case in which AB diffuses. Since nothing is promoting the production of A and B, their concentration will inevitably decay to zero and since AB diffuses its concentration on the long-term will inevitably become homogeneous (irrespectively of which initial pattern there may be).

Case in which only A diffuses. In this case the spike of A will initially lead to a valley of B (since A consumes B) and a peak of AB (since this consumption leads to AB). However, since for each molecule of AB a molecule of both A and B are required and the concentration of B is homogeneous (since B does not diffuse), having more of A around the spike would not lead to more AB in the peak than elsewhere. The concentration of AB would thus become homogeneous over time. The same applies if B is the only molecule that diffuses.

Case in which A and B diffuse and AB does not. In this case the spike of A will lead to a peak of AB. This would deplete B around the spike but since B can diffuse, new molecules of B would arrive and lead to a further growth in the peak of AB. As a result a stable peak of AB will form (even if A and B will ultimately decay to zero), just around the initial spike of A and both A and B will decay to zero (so no new peaks or valleys form and thus, no non-trivial pattern transformation).
